# Domestic burglary drop and the security hypothesis

**DOI:** 10.1186/s40163-017-0064-2

**Published:** 2017-02-15

**Authors:** Andromachi Tseloni, Graham Farrell, Rebecca Thompson, Emily Evans, Nick Tilley

**Affiliations:** 1 Quantitative and Spatial Criminology Research Group, School of Social Sciences, Nottingham Trent University, 50 Shakespeare Street, Nottingham, NG1 4FQ UK; 2 Centre for Criminal Justice Studies, School of Law, The Liberty Building, University of Leeds, Leeds, LS2 9JT UK; 3 School of Medicine and Health Sciences, University of Nottingham, Wollaton Road, Nottingham, NG8 1BB UK; 4 Department of Security and Crime Science, University College London, 35 Tavistock Square, London, WC1H 9EZ UK

**Keywords:** Crime drop, Domestic burglary, Security hypothesis, Data signatures, Crime Survey for England and Wales

## Abstract

This study examines the role of household security devices in producing the domestic burglary falls in England and Wales. It extends the study of the security hypothesis as an explanation for the ‘crime drop’. Crime Survey for England and Wales data are analysed from 1992 to 2011/12 via a series of data signatures indicating the nature of, and change in, the relationship between security devices and burglary. The causal role of improved security is strongly indicated by a set of interlocking data signatures: rapid increases in the prevalence of security, particularly in the availability of combinations of the most effective devices (door and window locks plus security lighting); a steep decline in the proportion of households without security accompanied by disproportionate rises in their burglary risk; and the decline being solely in forced rather than unforced entries to households. The study concludes that there is strong evidence that security caused the decline in burglary in England and Wales in the 1990s. Testing the security hypothesis across a wider range of crime types, countries and forms of security than examined to date, is required both to understand the crime drop and to derive lessons for future crime prevention practice and policy.

## Background

Sustained crime falls across a wide array of offences began in the United States in the early 1980s and in many other countries from the early 1990s. These have been referred to as the international crime drop (Tonry 2014; van Dijk et al. 2012) with the suggestion that there may have been a global crime drop (Tseloni et al. 2010). The widespread falls in crime came as a surprise to criminology (Farrell et al. 2008) and have posed a major challenge to those interested in understanding crime trends (Tonry 2014).

Reflecting the earlier crime downturn in the United States, early efforts to explain the crime drop stressed distinctive developments there (Blumstein and Wallman 2000). At least seventeen explanations have been identified in academic studies to date (Farrell et al. 2010; Farrell 2013). Due to their singular focus on the United States and inapplicability elsewhere, many of the early frontrunners have been discounted and categorised, with the benefit of hindsight, as parochial (Farrell et al. 2014; Tonry 2014). Most of the others appear to be contradicted by a range of specific evidence as well as the fact that they lack consistency with broader sets of evidence: They are inconsistent with the fact that crime rose for several decades previously; that some crime types, such as cyber-crimes and theft of some electronic products, have increased; and that there was significant variation in the timing and trajectory of crime declines both between and within countries (Farrell et al. 2014; Tseloni et al. 2010). The surviving hypothesis is the ‘security hypothesis’ (Farrell et al. 2011a): The crime drops are a function of reduced opportunities, which have been largely brought about by increases in the extent and quality of security, an idea first introduced by Clarke and Newman (2006) and by Van Dijk (2006).

The security hypothesis is underpinned by the crime opportunities theoretical framework of rational choice and routine activities. The link between crime and crime opportunities, and the role of security in their reduction, has long been established (Mayhew et al. 1976). The security hypothesis suggests that the infusion of everyday life with increasingly well-designed, unobtrusive and publicly acceptable security has led to the substantial crime drops that have been observed (see Tilley et al. 2015a). In addition there may well be some collective effects beyond the operation of individual devices, especially if potential offenders no longer assume that they can easily commit some types of crime with low risks of apprehension. Indeed increased security is the most common reason for the crime drop according to offenders interviewed in the four Australian states of New South Wales, Queensland, Western and South Australia (Brown 2015a). This fits with the rational choice theoretical perspective, according to which security improvements can be expected to increase the actual or perceived risk and effort of committing crimes or reduce the actual or perceived reward from them (Clarke 2012).

The security explanation of the crime drop is compatible with evidence that (a) different crime types have fallen at different times in different places, reflecting variation in how improved security has spread, and (b) particular types of security measures affect crime patterns differentially, producing distinctive crime-change ‘signatures’ (Farrell et al. 2014; Tseloni et al. 2010). Indeed, one of the advantages of the security hypothesis over other explanations of the crime drop is that it does not assume that all crime has dropped. It would expect crime increases where new crime opportunities emerge. Any new developments may inadvertently create new crime opportunities whose inhibition has not been built in from the start (Pease 1997), especially for CRAVED goods (concealable, removable, available, valuable, enjoyable and disposable) (Clarke 1999; Ekblom and Tilley 2000).^1^ This appears to have been the case with mobile phone theft (Thompson 2014; Office for National Statistics [ONS] 2013a) and cybercrime (McGuire and Dowling 2013). Thus, interspersed with an overall crime drop for many crimes produced by security increases, the security hypothesis would expect rises in some specific crimes where new opportunities have been created.

The diversity and ubiquity of security improvements create, however, huge challenges for testing the hypothesis as a whole. The task is that of specifying hypotheses that can be tested retrospectively with the available data, following a crime type—and country—specific approach (Farrell et al. 2008, 2010). Efforts to test the security hypothesis so far have focused on car crime. Car crime has fallen dramatically and there is mounting cross-national empirical evidence (based on available data that relate to the devices fitted to the vehicle itself) that central door locking, alarms and electronic immobilisers are especially important contributors to the drops in theft of and from vehicles (Bässman 2011; Brown 2013; Farrell et al. 2011b; Fujita and Maxfield 2012; Kriven and Ziersch 2007; Van Ours and Vollaard 2016). That said, it has to be acknowledged that these do not exhaust the ways in which the security of cars may have been increased.^2^ To our knowledge, the only previous study that touched upon longer term domestic burglary trends^3^ and house security comes from the Netherlands: Vollaard and van Ours (2011) conducted a cost-benefit analysis of a government regulation requiring burglary-proof windows and doors in new housing. They established that the built-in security in new homes reduced their burglary risks by 26% per year. It also contributed a net 5% to the overall burglary drop in that country in the decade following the regulation’s introduction.^4^

The present study contributes to research on the security hypothesis with a particular focus on domestic burglary in England and Wales. The security interventions to prevent domestic burglary have been unsystematic and non-universal: with the adoption of secured by design, some have been building age related, such as in the Netherlands study above, while others have been retrofitted to older properties. By contrast, the security interventions against vehicle crime were universal, implemented at the vehicle production phase and rolled out incrementally with vehicle age.^5^ Therefore this work moves the discussion on from car crime to explore the security hypothesis in relation to the decline in domestic burglary. It explicitly examines the security hypothesis over a longer period and a wider array of security combinations than previously, as well as with respect to burglars’ modus operandi. Thereby it expands crime signatures analysis to accommodate the challenges due to the different nature of the security interventions in residential properties compared to cars (Farrell et al. 2011b). We argue that the increases in availability of household security devices, coupled with an increased efficacy of security devices, provides a compelling explanation for the decline in burglary in England and Wales since the mid-1990s.

The next section discusses five key research hypotheses which test the effect of security on domestic burglary falls. An overview of the data and analytical strategy of this study follows. The "Findings" section is organised in five subsections each corresponding to a research hypothesis. Thereafter the theoretical and policy implications of the findings are discussed. Recommendations for further research and replications for testing the security hypothesis for the crime drop conclude this study.

## Domestic burglary and the security hypothesis

The remainder of this paper focuses specifically on the security hypothesis as it relates to domestic burglary in England and Wales. The research hypothesis is that improvements in security have played a major part in producing widely observed drops in burglary. Specifically the following changes would be expected if security has been important in producing the fall in domestic burglaries:

1. An increase in overall levels of household security.
2. A decrease in the proportion of households with no security and an increase in their relative burglary risk.^6^
3. An increase in the installation of more efficacious security devices and combinations of security devices.
4. An improvement in the efficacy of the security devices that are fitted.
5. A much larger drop in burglaries that required that security be overcome than in those where no security had to be overcome.

If these changes are found they provide support for the overall security hypothesis as it relates to domestic burglary.

The analysis presented used data from a national victimisation survey that has been conducted since 1982. The survey is now called the Crime Survey for England and Wales (prior to 2012 it was known as the British Crime Survey), and for consistency will be referred to here as ‘CSEW’.

**Fig. 1:**
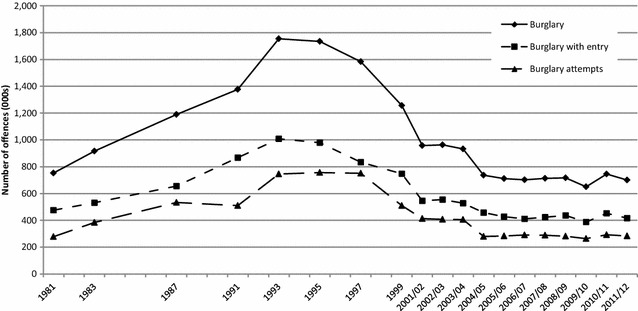
Trends in domestic burglary 1981 to 2011/12 (CSEW estimates). The CSEW data cover the calendar year, January to December, until 1999. Since 2001/02 they refer to the financial year, April to March. Source: Office for National Statistics (2013a) Crime Statistics, Focus on Property Crime, 2011/12. p. 16

Figure 1 shows the trend in domestic burglary from January 1981 to March 2012, as indicated from the CSEW. There has been a steep fall in burglary in England and Wales: numbers of recorded incidents dropped by 64% and the percentage of burgled households fell from 7 to 2.1 per 100 households between 1993 and 2011/12 (authors’ calculation from Table 11a, ONS 2013b: 55). Households are three times less likely to be burgled than they were in 1993 when CSEW burglary levels peaked. The sharpest burglary drop was between 1997 and 2001/02, at 39.5%—an average 10% per year during this short period (authors’ calculation from Figure 10, ibid.). Despite some fluctuations from year to year, the underlying trend has remained fairly stable since 2004/05, at around 700,000 incidents per year with non-statistically significant year-on-year variations.

Overall the figure indicates that both burglary with entry and attempts have dropped dramatically, although the fall in numbers of burglaries with entry began first. Attempts did not start falling until 1997, 4 years after ‘successful’ burglaries. The time lag between the beginning of attempts and burglary with entry falls indicates that burglaries fell due to target characteristics encountered after the target had been selected (such as unanticipated guardianship in the form of security) rather than offenders’ decisions not to target properties.^7^ Burglars’ ‘hit rate’ (burglary with entry over total number of burglaries) has fluctuated around 59% since 1981 and attained its highest value (63%) within the period of rising crime (1981 and 1991). However it reached its minimum of 53% the year attempts peaked, in 1997. It is worth noting that alongside burglary rates burglars’ ‘hit rate’ has remained fairly stable at 60% since 2004/05. In the remainder of this paper, the focus will be on burglaries with entry on the grounds that attempts may be thwarted by the presence of effective security devices, which the potential offender is unable to overcome, such as locks, or discouraged altogether by, say, internal lighting.^8^ Before turning to findings, as they relate to these issues, the CSEW data and methodology used in the analysis are described.

## The Crime Survey for England and Wales

This study’s evidence is based on analyses of sixteen CSEW sweeps, conducted between 1992 (referring back to events in 1991) and 2011/12, most undertaken in the course of the crime drop. The CSEW is a survey of the adult (16 years or older) population in England and Wales with currently 35,000 respondents per annum and consistently high response rates, between 73 and 83% (Jansson 2007; TNS-BRMB 2012). It is regarded as one of the most rigorous national crime surveys (Hough and Maxfield 2007).^9^ The CSEW, unlike police data, is not subject to changes in counting rules and offence categories, and therefore provides comparable year on year crime estimates (Van Dijk and Tseloni 2012).

The survey records crime experiences, including domestic burglary. Burglary victims are asked about details of incidents, including the security devices fitted to their properties at the time of the burglary.^10^ In addition, a randomly selected sub-sample is asked about the security devices fitted to their dwellings at the time of the interview. The security information provided by victims at the time of the burglary is a unique feature of the CSEW.

The number and type of security devices examined in the CSEW has improved slightly over time. Between 1992 and 1996, they included burglar alarms, double locks or/deadlocks, window locks and lights on a timer or sensor switch. Between 1998 and 2007/08 questions about dummy alarms, window bars/grilles and security chains were added, and lights were differentiated between indoor lights, and external lights on a timer or sensor. Between 2008/09 and 2011/12, CCTV cameras were added to the list. In order to obtain adequate sample sizes that enable meaningful statistical analysis of the large number of security combinations generated by the above list, the CSEW sweeps were merged: 1992–1996; 1998–2000; 2001/02 to 2004/05; 2005/06 to 2007/08 and 2008/09 to 2011/12. The Appendix provides details of the data sets, methodology and the security devices examined for both victims and non-victims of burglary over time.

## Analysis

This section places the analytic approach used in the study in a broader methodological context. Strong research designs are most straightforward where a single independent variable can be introduced in controlled conditions to observe its effect, if any, on the dependent variable to test the conjectured causal relationship. In relation to the security hypothesis, a different approach is necessary. The crime drops have occurred and the task is that of specifying hypotheses that can be tested retrospectively with the available data, with statistical confidence in the results. The analysis here does not describe a randomised experiment. Instead it relies on data describing contrasts between ‘treatment’ and ‘control’ samples which occurred in an unstructured manner, both gradually out of landlords’ and home owners’ own initiative and with regards to secured by design as a result of discrete changes in policy.

The CSEW data on security installed both at victims’ homes at the time of burglary and non-victims’^11^ residences at the time of interview delineate a quasi-natural experiment contrasting burglary risks between households with and without security (Dinardo 2010). This allows testing the security hypothesis via examining any causal effects of security on burglary. Since the cause, security, cannot be manipulated a quasi-natural experiment is not literally an experiment (Shadish et al. 2002). The methodology used to estimate the magnitude and statistical significance of the contrast between households with individual security devices and suites of them and those without security is the security impact assessment tool (SIAT) originally developed to test the effectiveness of car security devices (Farrell et al. 2011b).

The burglary risk for households without security is compared to the risk for households with a particular security device or combination of devices (both with respect to overall risk). The resulting metric, which is termed the security protection factor (SPF), shows how much less (or more) vulnerable a target is with given security devices compared to those with ‘no security’.^12^

Drawing on the above contrast provided in the CSEW, the present study develops a series of data signatures, whereby crime changes are consistent with expected outcomes from distinct context mechanisms outcome pattern configurations (CMOCs, Pawson and Tilley 1997). Having said this examining context, delineated by area and household type—specific patterns of burglary risks and security uptake trends, is beyond the scope of the current work.^13^ The methodology, which was originally developed to evaluate policy interventions using a realist evaluation approach, has acquired prominence in studies evaluating situational crime prevention interventions (Pawson and Tilley 1997). Each data signature is a discrete piece of empirical evidence that comprises a component of an overall triangulation approach to evaluation. As Eck and Madensen (2009: 69) highlight ‘[s]ignature changes consistent with expected intervention mechanisms eliminate rival explanations’ whereas those which are ‘inconsistent with the expected intervention mechanism undermine the validity of the conclusion that the intervention produced the crime change.’ The closer an observed outcome follows the expected pattern from the activation of the preventive mechanisms and the fewer alternatives exist, the more confident we can be in attributing causality to it (Pawson and Tilley 1997). The five expected changes outlined in the "Domestic burglary and the security hypothesis" section provide the data signatures pointing to the pivotal role of security in domestic burglary falls.

## Findings

This section tests the five research hypotheses presented earlier, one at a time. Collectively these hypotheses illustrate the patterns expected if security measures were to have played a major part in domestic burglary with entry fall shown in the data section.

1. Was there an increase in overall levels of household security?

**Fig. 2:**
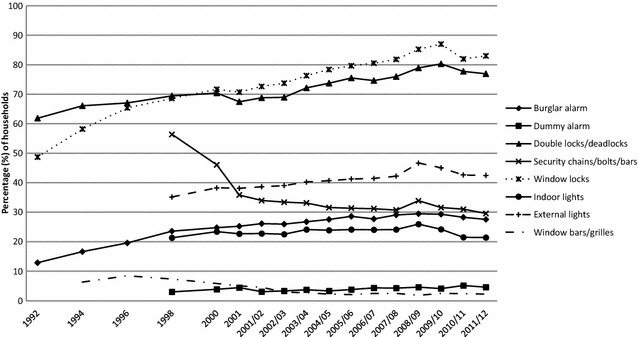
Trends in the availability of burglary security devices, 1992–2011/12 CSEW

Figure 2 shows that between 1992 and 2011/12 there was a general increase in the proportion of households fitted with a range of security devices which preceded the burglary falls (see earlier Fig. 1). Window locks were fitted to a little less than 50% of all households in 1992, but were fitted to a peak of 87% in 2009/10. Likewise double locks/deadlocks were fitted to external doors in just over six in ten households in 1992, but to around eight in ten by 2009/10. Burglar alarms were fitted to slightly more than 10% of households in 1992, but close to three times as many by 2008/09. With the exception of *security chains* (which halved from 56.38 to 29.59%) all popular^14^ security devices became widespread. However, combinations and the number of devices are not shown in Fig. 2. All that can be seen are trends in the proportion of households with each device without reference to the presence of any other device. Therefore the proportion of households with each device alone or any possible combination fitted has to be specified.

**Fig. 3:**
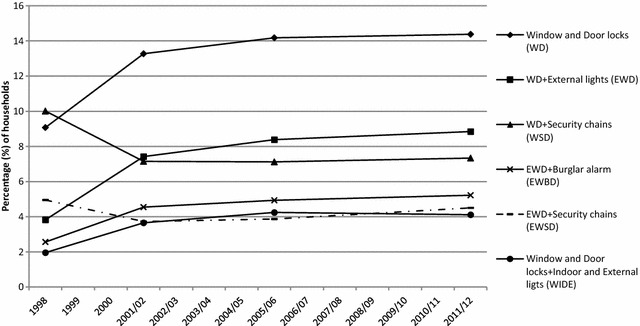
Most common combinations of security devices in households in England and Wales, 1998–2011/12 CSEW

Figure 3 shows the most popular combinations fitted in households across the four periods of merged CSEW data, starting from 1998 to 2000—the period of sharp drop (see Fig. 1).^15^ With the exception of combinations including security chains, the proportion of households fitted with more than one device increased. Households with window locks and double or deadlocks on doors as their only security present and those having also external lights roughly doubled (from 8 and 4% in 1998–2000 to nearly 15 and 9% in 2008/09 to 2011/12, respectively). Moreover the steepest rise in window and door locks (and external lights) occurred between 1998 and 2001/02, the period of the sharpest burglary fall. The combination of window locks and double or deadlocks on doors remain the most popular security devices for households, perhaps linked with insurance incentives or the spread of energy-saving storm windows (that is, double glass panels known as ‘double glazing’ in England and Wales).

2. a. Was there a decrease in the proportion of households with ‘no security’? and
  b. Did their relative burglary risk rise?

**Fig. 4:**
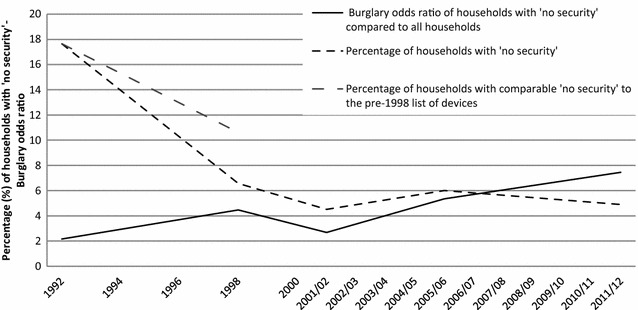
‘No security’ burglary with entry trends 1992–2011/12 Crime Survey for England and Wales. The definition of ‘no security’ alters after 1992–1996, as the range of security devices asked about in succeeding batches of sweeps changes. The original ‘no security’ for 1998 is shown in the* grey dotted line*. Burglary odds ratio refers to 1991, 1993, 1995, 1997 and 1999 and from 2001/02 the years indicated in the* x-axis*

The prevalence of households with ‘no security’ and their burglary risk relative to overall burglary risk is presented in Fig. 4. There was a major fall in the proportion of households with ‘no security’, reducing the supply of properties where none of the listed security devices are in place to make burglary with entry more difficult or risky. Households with ‘no security’ declined by 72% (from 17.65 to 4.90%) between 1992 to 1996 and 2008/09 to 2011/12. The sharpest decline of around two-thirds of households with ‘no security’ measures occurred in the period 1992–1998 which directly preceded the sharpest burglary falls of the years 1997–2001/02 (Fig. 1). Although the decline between 1992–1996 and 1998–2000 is partly an artefact of the increase in CSEW listed devices (the top line in Fig. 4 shows a respective 40% reduction in households with ‘no security’ as defined in the earlier than the 1998 CSEW), the proportion of households with ‘no security’ continued to fall after 1998 and remained low to around 5% for the remaining years.

Households with ‘no security’ are known to be at very high risk of burglary with entry (Budd 1999) and Fig. 4 confirms this. Households with ‘no security’ were twice as likely as the general population to be burgled in 1992–1996 and nearly eight times more so in 2008/09 to 2011/12. Therefore households with ‘no security’ have experienced a fourfold increase in their relative burglary risk during the crime drop and especially since 2001/02. This agrees with the overarching assumption that security was the main driver for the burglary falls.

Another hypothesis that builds upon the second part of the hypothesis addressed here is that there is more security among those targets that have seen the greatest decline in burglary (see footnote 5). Indeed, the crime fall was uneven across different population groups and areas and property crime concentration has increased during the crime drop (Ignatans and Pease 2015, 2016). Owner occupiers, for example, whose homes have more security than rented accommodation, benefitted the most from burglary falls (Hunter and Tseloni 2016; Tseloni and Thompson 2015). However examining this is beyond the scope of the current paper as it deserves to be addressed in a separate piece work (see footnote 13).

3. Was there an increase in the installation of more efficacious security devices and combinations of security devices?

Some security devices are more effective than others in reducing the risk of burglary with entry based on their SPFs from the 2008/09 to 2011/12 CSEW data (Tseloni et al. 2014). Moreover, a greater number of security devices is generally more effective than fewer although the benefit of more than four devices is negligible. The most effective combination of two devices is window locks and door double or deadlocks (SPF = 13); the most effective combination of three involves adding external lights on a sensor (SPF = 34); and the most effective four adding internal lights on a timer (SPF = 49).

The protection conferred by suites of devices generally exceeds the sum of the impact of the separate devices. The value added from combining individual devices is given by the Net Interaction Effect (NIE) of security configurations. For example, properties fitted with the four ‘WIDE’ devices: window locks (W), indoor lights on a timer (I), door double or deadlocks (D) and external lights on a sensor (E) have an SPF of 49. The sum of the SPFs of the individual devices (W = 7, I = 3, D = 3 and E = 3) is, however, only 16. The WIDE NIE is, therefore, 33 (calculated as 49 − 16). Of the 41 security suites examined, 28 had a positive NIE for burglary with entry. Five had zero NIEs and only eight had negative NIEs, of which more will be said later (Tseloni et al. 2014).^16^

**Fig. 5:**
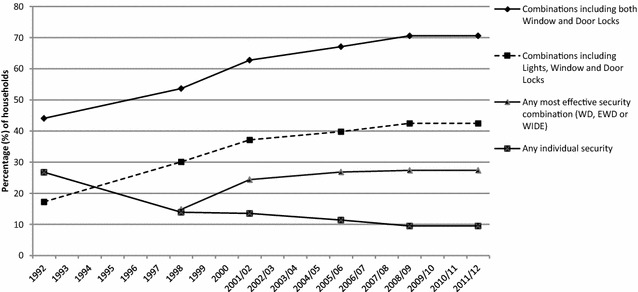
Efficacious security combinations of devices and single devices in households in England and Wales, 1992–2011/12 CSEW. The 1992–1996 CSEW data about lights is assumed to correspond to external lights, internal lights or both in the post-1996 CSEW sweeps

Figure 5 shows the trajectories of security uptake for comparable efficacious combinations and single device presence during the period examined. The proportion of households with any of the three most efficacious security combinations mentioned earlier (WD, EWD or WIDE) has almost doubled (84%) from 1998 (when comparable data exist) to 2011/12. From 1992–1996 to 2008/09–2011/12 the proportion of households with any security configuration that included window and door locks rose by 60%. Those with configurations of both locks and any lights (for comparability with the pre-1998 data) more than doubled (146%). The steepest increases in efficacious security combinations had already occurred by 2001/02 in the period before and during the sharpest burglary fall. By contrast, there were almost two-thirds (64%) fewer households with just one device at the end of the period examined compared to 1992–1996. The greatest decline occurred just before and at the start of the burglary falls (1992–1998).^17^ Summing up there has been a substantial increase in multiple security (also seen in Fig. 3) and especially in high efficacy combinations before, at the start and during the sharpest (10% annually) burglary drop in England and Wales (1992–2001/02), accompanied by a speedy reduction in households with no (Fig. 4) or single device security in the period around the beginning of the fall (1992–1998).

4. Was there an improvement in the efficacy of the security devices?

**Fig. 6:**
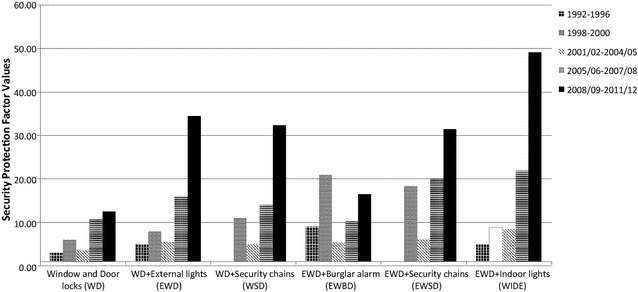
Changing SPFs for selected security combinations, 1992–1996 to 2008/09 to 2011/12 CSEW. The 1992–1996 CSEW data about lights is assumed to correspond to external lights, internal lights or both in the post-1996 CSEW sweeps. The* white bar* for 1998–2000 WIDE denotes non-statistically significant odds ratios and SPF. Security was measured at the time of the interview for non-victims and the time of burglary for victims

Clearly, window and door locks can be stronger or weaker, alarm systems can be more or less extensive or sensitive and may or may not be connected to monitoring stations, and lighting arrangements can vary in their intensity and responsiveness to movement. However, the CSEW has asked only about the presence or absence of security devices. It has not been designed to capture their quality, nor whether the devices were in use at the time of the victimisation. Nevertheless, if security has played a part in the crime drop, given that security measures appear to be effective in inhibiting burglary and given that quality varies, it is important to try to gauge whether their effectiveness had increased in ways that help explain the fall in burglary with entry. To try to estimate changes in efficacy we calculated the SPFs for burglary for the successive sets of CSEW data from 1992 to 2011/12. The results for the most frequently installed security combinations (see Fig. 3) are shown in Fig. 6.

With one exception the trends are clear: The protective power of all commonly installed suites of security devices, increased exponentially over time even more than their presence (see Figs. 3, 5). This is suggestive of increased efficacy (and implied quality improvements) and together with the previous discussion explains why burglary fell sharply while security did not expand to more households after 1998 (see Fig. 4). Notwithstanding the security hypothesis however the greatest ‘efficacy’ gains happened after 2004/05, when burglary rates plateaued. This implies an uneven take up of efficacious security and large variations in burglary trends across areas and population groups (as already seen in the increasing burglary risk of ‘no security’ households and the discussion of Fig. 4) which since 2004/05 have averaged out nationally (see footnote 5).

**Fig. 7:**
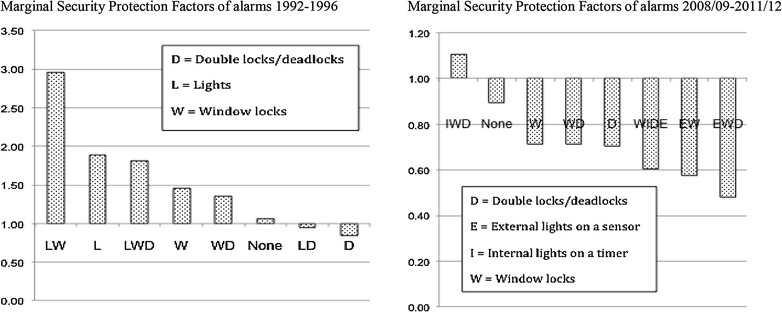
Marginal security protection factors from the addition of burglar alarms to selected comparable security combinations: 1992–1996 and 2008/09 to 2011/12 Tilley et al. (2015b)

The exception of increasing protective power over time refers to burglar alarms: The effectiveness of the security combination that incorporates a burglar alarm (EWBD) in Fig. 6 declined over time. Moreover adding a burglar alarm (EWBD) to the combination of window locks, door double or deadlocks and external lights (EWD) reduces its efficacy for all years after 2000. It looks as if the addition of the burglar alarm to the EWD configuration of security devices led to less rather than more protection from burglary with entry since 2001/02.^18^ Figure 7 shows whether the addition of an alarm increases or decreases security for two sets of merged CSEW sweeps, 1992–1996 and 2008/09 to 2011/12, across comparable security configurations.

The addition of an alarm resulted in a consistent increase but a consistent decrease in security in 1992–1996 and 2008/09 to 2011/12, respectively, for the examined combinations.^19^ The paradoxical findings for EWD and EWBD in 2001/02 to 2011/12 are therefore part of a more general pattern and not exceptional nor confined to England and Wales (Bettaïeb 2015; ICPC 2015). From the various possible explanations for alarms’ ineffectiveness leading conjectures include the changing balance between risks and gains for the prospective burglar that alarms signal and/or alarms normalisation for home/contents insurance coupled with poorer average quality over time (Tilley et al. 2015b). Further research is needed but what the findings so far suggest is that it would be unwise to assume that, although more security devices generally increase security, this is invariably the case.

Therefore over time security has become more widespread and, with the exception of burglar alarms, works better in deflecting burglaries. The results provide strong evidence that increasing adoption of efficacious suites of security devices produced growing protection from the risk of burglary with entry and are consistent with the security hypothesis.

5. Was there a much larger drop in burglaries that required that security be overcome than in those where no security had to be overcome?

**Fig. 8:**
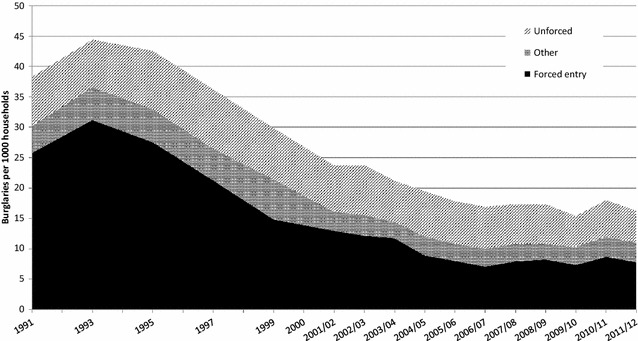
Burglaries per 1000 households by forced and unforced entry, 1991–2011/12 (CSEW estimates)

The CSEW asks victims of domestic burglary a series of questions about the nature of the incident, relating to how the offence was committed and whether anyone was at home at the time it took place. Changes in the rate and distribution of these can be used as indicators for the relevance of security to the drop in burglary with entry. Specifically, if changing availability and quality of security were important in driving down burglary with entry it would be expected that the drops in burglary would be concentrated amongst those that required that security be overcome. Figure 8 shows trends of burglary incidence rates from 1991 to 2011/12 distinguishing across burglars’ modus operandi: *forced entry*, which involves forcing lock or window, breaking/cutting glass or breaking/removing door panel; *unforced entry*, which includes entering via doors or windows that were left open or unlocked; and *other*, such as burglar(s) having a key, pushed past, or entering by false pretences and other entry. The rate of burglary involving forced entry methods has dropped dramatically, while that using modus operandi involving unforced entry has remained relatively stable. For example, burglaries with forced entry per 1000 households dropped from 31 in 1993 to 7 in 2006/07, a fall of 77%, while unforced entry burglaries remained at 7 per 1000 households in the same period.

**Fig. 9:**
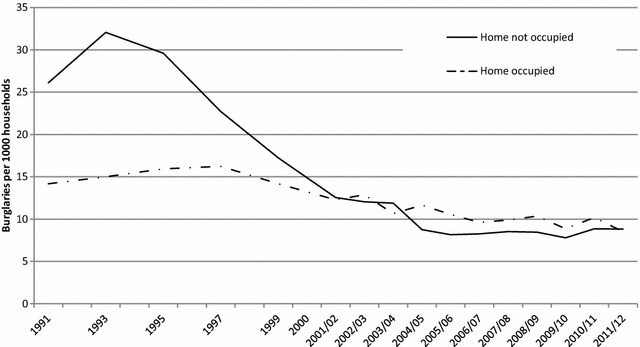
Burglaries with entry per 1000 households by whether home was occupied at the time of the incident, 1991–2011/12 (CSEW estimates)

Given that many security devices are activated (such as closing and locking doors and windows, priming alarms and switching on security lights) when a dwelling is left empty, if security devices are effective this should, in theory, be manifested in greater falls in burglary with entry when no-one is at home. Figure 9 indicates that the main drop in burglary has occurred where the house is empty rather than when it is occupied. The data signatures delineated in Figs. 8 and 9 confirm the patterns expected if the security hypothesis is correct.

## Discussion

The purpose of the research reported here was to extend the empirical test of the security hypothesis as an explanation for the widespread crime drop. To do so it examined the relationship between domestic burglary, household security devices and burglars’ modus operandi over time based on data from the CSEW. The findings support the hypothesis while they do not find that all devices were similarly responsible. The use of combinations of security devices appears to have been particularly important, especially door and window locks plus security lighting while, somewhat counterintuitively, alarms were not identified as contributory. The findings reported here align well with the positive findings for the security hypothesis reported earlier in relation to theft of and from vehicles cross-nationally and domestic burglary in the Netherlands.

The findings, however, reflect the limitations of the available data, excellent though the crime survey drawn on is. For example, the proportion of households with storm windows (double glazing) to all external doors and windows more than doubled while the rate of burglary with entry more than halved between 1996 and 2008 (see Tilley et al. 2015a). Moreover, some security measures are targeted at an area rather than individual households, the unit of analysis here. These include secured by design (SBD) housing planning and construction standards; levels of ambient lighting sometimes increased in the interests of crime prevention; alley-gating to restrict access to the backs of properties where covert access may otherwise be readily obtained; and the widespread use of and publicity for DNA-like property marking. The implementation of SBD planning and building standards, first introduced in the UK in 1989, greatly expanded from 1998 (Armitage and Monchuk 2011) which coincides with the sharpest fall in burglaries (between 1997 and 2001/02, Fig. 1). The relationship of all the above changes however with the burglary falls cannot be tested with CSEW data. Consequently, it is quite possible that the overall role of security, when more broadly defined, is understated by the present study.

The data do not speak to mechanisms through which the inhibition of some volume crimes may multiply preventive outcomes. These mechanisms include, for example, the inhibition of the onset of criminal careers by removing the easy opportunity for novice offenders to become criminally involved with their associates (the debut crime hypothesis) or the removal of easy opportunities to raise the money needed for purchasing illicit drugs and becoming habituated to them in ways that foster further criminal behaviour. Testing whether these indirect crime-inhibiting out-workings of security induced falls in common acquisitive crimes lies beyond the scope of this study and provides avenues for future research on the security hypothesis.^20^ Other suggestions for expanding this work include context (reflected in area or household characteristics)-based analysis of the relationship (and the distributional justice) between security and crime trends; cost-benefit analysis (including carbon footprint costs) of fitting security; and investigating any diminishing marginal return of security in low risk areas (see footnote 5).

The research findings reported here have direct practical implications for maintaining or extending the crime drop. Previous research has shown that security is unevenly distributed across the population (Tilley et al. 2011; Tilley 2012; Tseloni and Thompson 2015). Moreover, the poorest sectors of the population and those who rent property privately or from local authorities (in the USA public housing tenants) are amongst the most at risk of domestic burglary (Hunter and Tseloni 2016; Tseloni and Thompson 2015). Those who have already been victimised are the most vulnerable of all and previous burglary victimisation is the single highest predictive factor of current risk (Farrell and Pease 1993; Osborn and Tseloni 1998). The clear finding that WIDE security devices (Window locks, Indoor lights on a timer, door double or Deadlocks and External lights on a sensor) produce a very substantial (almost 50 times) lower risk of burglary with entry than ‘no security’ suggests that these devices be targeted on the most vulnerable. Targeting could take the form of advice by police, insurance companies or government, subsidies for the installation of devices by charities or crime reduction partnerships, or legislation to decree that these devices be required as minimum standards to be met by property developers and by landlords for their tenants. The findings also suggest rethinking advice regarding burglar alarms. It would be prudent to think twice before recommending an alarm as part of a standard package of security devices to reduce the risk of domestic burglary. Pending further research to understand better why alarms are associated with increased rather than decreased risk of burglary with entry, the requirement by insurance companies that alarms be fitted as a condition for continued coverage or for avoiding increased premiums becomes questionable.

## Conclusion: where next?

The empirical evidence reported here lends support to the security hypothesis as an explanation for the crime drop. Research to date has largely established the critical role of vehicle security in reducing car crime cross-nationally. The present study, drawing on triangulation of data signatures from sixteen sweeps of national victimisation surveys covering the period 1991–2011/12 that describe a quasi-natural experiment, shows that increases in the prevalence and effectiveness of house security have been a major driver of the domestic burglary falls in England and Wales. Therefore it expands coverage of the security hypothesis quite significantly.

The security hypothesis is important not only because it relates to the major question currently facing criminology, ‘Why did the long term trend of increasing crime reverse?’, but also because of its clear implications for crime prevention practice and policy. If security has been largely responsible for the massive and unexpected falls in past volume crimes, including burglary and theft of and from vehicles, crime policy should focus on reducing or pre-empting crime opportunities for new and emerging volume crimes, perhaps most notably cyber-crime, that have been facilitated by the internet.^21^

The results presented here do not *prove* the security hypothesis for the international crime drop. First, they relate to one crime type in one jurisdiction. Second, they rely on data that do not describe a randomised but a quasi-natural experiment, studying the contrasts recorded by the CSEW, a very strong series of victimisation surveys, between households with and without security devices. Indeed, the available data have been analysed to determine whether the precisely expected patterns that can be elicited are congruent with the security hypothesis. Therefore it has not been possible to control for all changes that have occurred. For example, ideally changes in the quality of security devices, and/or area-based security improvements ought to have been measured and tested in relation to producing the overall drop in burglary. Likewise the research reported here for England and Wales could usefully be replicated in other countries where there has been a drop in burglary (or where, conversely, burglary has risen) to ascertain whether similar household security improvements (or relaxing of security) have been made elsewhere with a similar role in producing drops (or rises) in burglary. Therefore while the present study comprises a step in understanding whether and how security has contributed to the drop in burglary, there is more work to be done.

While there is room for doubt we conclude that for burglary with entry the evidence supporting the primary role of security is now beyond reasonable doubt. This evidence also concurs with that for vehicle crime. We suggest it is now up to others to find evidence that could falsify the security hypothesis as it relates to burglary and vehicle crime or to look to evidence in other jurisdictions and for other crime types to determine whether similar findings suggest likewise that security improvements have led to widespread falls in crime.

Although the security hypothesis has been tested in relation to some specific crimes, what is observed are drops in many types of crime across many countries. Yet wide-ranging security developments, which Clarke (2016) refers to as an avalanche of security, now wash through much of everyday life. There is a need to catalogue those security improvements, establish where and when they were introduced, and to identify and assess the relevant crime pattern change signatures. There is also a need for further research into whether security brought down violence either directly or indirectly. There is, in short, significant potential for further research into the security hypothesis.

## Appendix

### Data and methodological clarifications

To increase the potential number of homes with any possible security configuration from the consistently available list of devices (see Table 1) adhering also to changes in this list and the survey’s sampling design, the various sweeps of the CSEW data have been merged to provide five composite data sets as follows:

**Table 1:** Household security measures measured by the CSEW in both the victim and non-victim questionnaires, 1992–2011/12

	1992	1994	1996	1998	2000	01/02	02/03	03/04	04/05	05/06	06/07	07/08	08/09	09/10	10/11	11/12
Burglar alarm	✓	✓	✓	✓	✓	✓	✓	✓	✓	✓	✓	✓	✓	✓	✓	✓
Dummy box				✓	✓	✓	✓	✓	✓	✓	✓	✓	✓	✓	✓	✓
Double locks/deadlocks	✓	✓	✓	✓	✓	✓	✓	✓	✓	✓	✓	✓	✓	✓	✓	✓
Security chains/bolts				✓	✓											
Security chains						✓	✓	✓								
Security chains/doorbars									✓	✓	✓	✓	✓	✓	✓	✓
Window locks	✓	✓	✓	✓	✓	✓	✓	✓	✓	✓	✓	✓	✓	✓	✓	✓
Indoor lights				✓	✓	✓	✓	✓	✓	✓	✓	✓	✓	✓	✓	✓
Outdoor lights				✓	✓	✓	✓	✓	✓	✓	✓	✓	✓	✓	✓	✓
Lights	✓	✓	✓													
Window bars/grilles		✓	✓	✓	✓	✓	✓	✓	✓	✓	✓	✓	✓	✓	✓	✓
CCTV camera													✓	✓	✓	✓

1. 1992, 1994 and 1996 CSEW data formed the 1992–1996 data set with reference period from January to December 1991, 1993 and 1995;
2. 1998 and 2000 CSEW data formed the 1998–2000 data set referring to January to December 1997 and 1999;
3. 2001/02, 2002/03, 2003/04 and 2004/05 CSEW data formed the 2001/02 to 2004/05 data set with continuous reference period from April 2001 to March 2005;
4. 2005/06, 2006/07 and 2007/08 CSEW data formed the 2005/06 to 2007/08 data set with continuous reference period from April 2005 to March 2008; and
5. 2008/09, 2009/10, 2010/11 and 2011/12 CSEW data refer to the period from April 2007 to March 2012.

When a victim reported repeat burglary incidents via more than one long Victim Form their home security availability at the time of the *first* burglary during the survey’s reference period has been retained for analysis^22^ since the unit of analysis is the household.

The SIAT methodology requires information about security devices for both the general population and burgled households. Table 1 indicates the precise home security devices that were examined in both the Victim Forms and the Crime Prevention module from 1992 to 2011/12.

Strictly speaking ‘no security’ is non-comparable over time except between the 1998 and the 2007/08 CSEW sweeps. ‘No security’ in the 1992–1996 sweeps means no burglar alarm, no double locks, no window locks and no lights. From the 1998 sweep onwards more questions were asked so in addition to the previous list, ‘no security’ means no security chains, no indoor timer lights and no external sensor lights. The fact that ‘no security’ in the earlier 1992–1996 CSEW sweeps means something different to ‘no security’ in the following sweeps may to some extent explain the higher frequency of ‘no security’ prior to 1998. As shown clearly in Fig. 4 however it does not negate the considerable fall of households with ‘no security’ from 1992 to 1998. In theory ‘no security’ is not fully comparable for the pre- and post-2008/09 sweeps of the CSEW due to the introduction of questions about the availability of CCTV camera in the respondent’s home. In practice the negligible prevalence of CCTV camera (0.15% of households) removes such concerns. Therefore ‘no security’ is comparable in principle during the period of substantial burglary falls, 1998–2007/08, and in practice from 1998 onwards.

The burglary risk for households without security is compared to the risk for households with a particular security device or combination of devices (both with respect to overall risk) as shown in the following equation:

$$\frac{Burglary\;risk\;no\;security}{Overall\;burglary\;risk }\Big/\frac{Burglary\;risk\;with\;security}{Overall\;burglary\;risk}$$

The resulting metric, which is termed the security protection factor (SPF), indicates the protection conferred by a security device or suite of devices compared to no security, with the distance of the obtained value from 1 indicating the impact in relation to burglary, as outlined below:

**Table:** Interpreting the security protection factor (SPF) metric

SPF value	SPF interpretation
>1	Security device(s) associated with a lower risk of burglary
1	Security device(s) have no discernable impact on burglary risk
<1	Security device(s) associated with a higher risk of burglary

The SPF shows how much less (or more) vulnerable a target is with given security devices compared to those with ‘no security’ (see footnote 12). A score of two would mean that targets with the security measure(s) in place are at half the risk or in other words twice as safe as targets with no security devices. A score of 0.5 would indicate that targets with the security measure(s) in place are half as safe or in other words face twice the risk as those targets with none of the listed security devices. Any score of over one, therefore, indicates less risk than a target with ‘no security’. Any with a score of less than one indicates more risk than a target with ‘no security’.

Combinations of devices usually offer greater protection than the sum of the SPFs of each device included in the combination. The Net Interaction Effect (NIE) of security configurations indicates the value added from combining individual devices. It is calculated by subtracting the sum of the SPFs of individual devices that make up each security combination from that of the combination. The findings discuss this further based on specific security combinations whereas Tseloni et al. (2014) provide complete results on the efficacy of security devices and their NIEs across all security combinations.

In order to determine whether the addition of a specific device more generally increases or decreases security, marginal SPFs can be calculated using the SIAT (Tilley et al. 2015b). The marginal SPFs compare the risk of burglary with entry of households with a given set of security devices including, say, a burglar alarm, with that same set without a burglar alarm. The results can be read in the same way as SPFs in the previous table: A score of more than one indicates increased protection or reduced burglary risk. A score of less than one indicates decreased protection from the addition of a burglar alarm to a constellation of security devices.

## Abbreviations

CMOC: context mechanisms outcome pattern configurations
CRAVED: concealable, removable, available, valuable, enjoyable and disposable
CRAAVED: concealable, removable, accessible, abundant, valuable, enjoyable and disposable
CSEW: Crime Survey for England and Wales
D: door double or deadlocks
E: external lights on a sensor
EWBD: external lights on a sensor, window locks, burglar alarm and door double or deadlocks
EWD: external lights on a sensor, window locks and door double or deadlocks
I: indoor lights on a timer
NIE: net interaction effect
ONS: Office for National Statistics
SBD: secured by design
SIAT: security impact assessment tool
SPF: security protection factor
W: window locks
WD: window locks and door double or deadlocks
WIDE: window locks, indoor lights on a timer, door double or deadlocks and external lights on a sensor

## Footnotes

1 Pires and Clarke (2012) have suggested that CRAVED might be updated with CRAAVED suggesting available could be replaced with accessible and abundant.

2 Many car parks have been made more secure via CCTV cameras, barriers and improved lighting, reducing the risk to cars parked in them (Webb 2005; Smith et al. 2003; Poyner 1992; Tilley 1993). Also, in Australia and England and Wales new car registration arrangements have made it more difficult to change the identity of a stolen car (Brown 2015b; Webb 2005).

3 A number of studies has examined the short term effects of target hardening and security policy initiatives, including Secured by Design evaluations, on domestic burglary (for example, Armitage and Monchuk 2011; Ekblom 1996).

4 The study did not examine the effect of other security devices or security fitted retrospectively in existing homes both of which may be responsible for the bulk of Dutch national burglary falls.

5 With thanks to the Anonymous Reviewer who pointed this out.

6 The first two hypotheses are distinct: the first refers to an increase in security devices per household and the first part of the second to more households with any sort (single or multiple) of security or less households without any security.

7 Simultaneous or preceding drop in attempts compared to burglary with entry would have indicated that burglars’ decisions not to target properties (perhaps due to low financial returns from this crime type) might have been a reason behind the burglary fall. However the ‘typical gain’ for an offender does not vary considerably before and after the crime drop. Moreover between 2000 and 2013/14, the average value of goods stolen through burglary *increased*, alongside a decrease in the number of incidents (Shaw et al. 2015).

8 Therefore security can exert both a positive and negative effect to attempts requiring security device—specific analyses to address the role of security on attempts falls.

9 For details on the CSEW methodology and questionnaire, see Hough and Maxfield (2007), Flatley (2014) and the various CSEW Technical Reports, which are accessible via http://www.ons.gov.uk/ons/taxonomy/index.html?nscl=Crime+in+England+and+Wales.

10 For repeat victims of domestic burglary the security of the first incident experienced is included in this analysis to gauge its preventive effect. An initial burglary increases the demand for and use of security devices (Budd 1999; Philipson and Posner 1996). However victims’ response to a first burglary and whether this alters subsequent burglary risk, whilst acknowledged as of great interest, is outside the scope of the present study.

11 Non-victims refer to those households which have not encountered any type of burglary. Attempts are not discussed in this paper but in work not reported here they have been analysed similarly to burglary with entry, so they are not removed from the analysis or subsumed with non-victims.

12 By ‘no security’ we mean respondents who stated they had none of the listed security devices. Therefore this is not directly comparable before and after 1998 as the security devices included in the CSEW change (see Appendix). However we attempt to adjust for this in Fig. 4 and the later Figs. 5 and 6.

13 Area type- and population group- specific estimates of the relationship between burglary risk and security during the crime drop is indeed beyond the scope of the current work and requires a different methodological approach to avoid omitted variables problems.

14 These are defined here as security installed in more than 10% of households.

15 The 1992–1996 set of data is omitted in Fig. 3. The CSEW sweeps have not asked about an identical range of security devices fitted to households before and after 1998 impeding comparisons of exact security combinations with the 1992–1996 period.

16 The calculations relating to NIEs here are based on rounded figures (Tseloni et al. 2014).

17 Any individual security excludes CCTV cameras which were available to only 0.15% of households as a single device in the period 2008/09 to 2011/12. Such negligible prevalence of CCTV cameras in households in effect does not influence the pre- and post-2008/09 comparisons of ‘no security’ and any suites of security devices. Overall (individually or in combination with other devices) 4.6% of households had CCTV cameras.

18 A full description of this work can be found in Tilley et al. (2015b).

19 The two panels in Fig. 7 need to be read with caution as the MSPFs are not precisely comparable (see Appendix).

20 These important for the security hypothesis research questions may draw on longitudinal criminal careers data.

21 We note that street crime did not switch to cyber-crime: the internet arrived too late and cyber-crime is a poor substitute for car theft and burglary (as argued by Farrell et al. 2015).
